# Influence of Diseased Teeth upon the Health

**Published:** 1872-03

**Authors:** N. E. Hollace

**Affiliations:** Boston, Mass.


### ARTICLE II

### *Influence of Diseased Teeth upon the Health.*

BY N. E. HOLLACE, M. D., BOSTON, MASS.

The bad effects of a diseased and unclean mouth upon the general health, are of a more serious consequence than most physicians are aware. In twenty-four hours we breathe twenty thousand times, and what must be the effect upon the delicate structure of the lungs, when for days, months, and years, the air we breathe is drawn through a depository of filth, and is poisoned by being mixed with effluvia arising from decayed teeth and ulcerated gums.

That diseased condition of the teeth and the structures adjacent to them, do exert a most pernicious influence upon the general health, is a fact as well established as any other medical observation; yet it is a sad, but notorious fact, that the great mass of practitioners do not appreciate as fully as they ought the importance of the teeth and their diseases. I am not aware that the subject is ever alluded to by lecturers on the practice of physic, when recapitulating to their classes the causes of functional disturbance and constitutional suffering; certainly, however frequently the physician may look into the mouths of his patients, it is very rarely that his comprehensive glance perceives anything worthy of note in the decaying organs of mastication.

It is fully time that practitioners of medicine should perceive the importance of the teeth, and of their diseases; but until they do so it is the more important that the dentist should be able to point out the causes of obscure disease, which the physician has in vain endeavored to discover, simply because he has sought for it everywhere but the right place.

It might be granted a priori, that as physiological conditions of the teeth, owing to their peculiar position, associations and history, may exercise powerful influence upon the health of other organs, pathological conditions of these same teeth cannot be entirely harmless. Again, if we would examine the structure of a tooth, and perceive how completely its sensitive part is enclosed in an unyielding bony case, we might readily infer from the consequences of compression in other parts that the swollen and inflamed pulp would be exceedingly painful.

If, too, we would regard the close connection existing between the teeth, the rapidity with which the flash of sympathetic pain darts along the nervous cords which vitalize them, and the intolerable and protracted suffering which ensues, upon even trifling irritation of these sensitive filaments, and remember that pain itself is fully capable of deranging the whole economy, and inducing serious and fatal disorder, we might without the aid of much reflection, adopt the very rational conclusion that the diseases of the teeth must be of considerable consequence to the entire organization.

We might also, with similar propriety, conclude that the teeth were not made merely for ornament, and that mastication and insalivation are something more than mere forms of introduction to the stomach; that they are important to digestion which is importance to the entireness of organs and the performance of function, and that if mastication and the insalivation accompanying it be imperfectly performed, some corresponding imperfection of digestion must result.

We might also infer from the known consequence of long continued morbid influences, however unimportant in their immediate action, that disturbance of digestion constantly repeated, must in time, develop evils of a serious character. The old pathological maxim, "where there is irritation, to that part will be the flow," is a valuable lesson to the medical practitioner. It is true that the nervous, and to a certain degree even the vascular forces hurry to the part which throws out the signal of distress, and all the floating energies of the system are directed to the relief of the suffering. If it can be readily accomplished, the equilibrium of the system is soon restored and no perceptible inconvenience results. But if from the impracticable nature of the tissue or organ affected, but little relief can be given, and if the efforts of nature to accomplish a cure or removal of the part, end only in accumulating about it an uncommon amount of sensibility increasing the irritation and demanding yet more of constitutional effort to combat it, the consequence must be such a diversion of nervous influences from other parts as to weaken their force of action and to embarrass their functions.

In short, it is easy to understand that when the first movement towards constitutional derangement has been made, if the cause continues to act, each accession of morbid condition must aggravate and extend the evil, and hence it is that causes in themselves very slight, may if long continued, from the influence of sympathy and the accident of relations, induce morbid conditions of the most serious character.

The extremely hard and dense structure of the bony parts of the teeth, and the great arterial activity and nervous irritability of their lining membranes, which can so powerfully and so long a time defend the teeth against general, local, and morbid influences, arealso causes of their producing very extensive morbid effects upon the whole system.

The bony structure of the teeth, however, having in itself but little self restoring power, and their peculiar functions being much less favorable to this natural process than those of any other part of the body The teeth, gums and periotium, being altogether dependent upon each other, this power is much more constantly, and in a much higher degree required, and seems to be much more exerted by these than by any other structures, and the more these powerful efforts are incapable of curing the dental diseases, and resisted in their efforts to remove their causes, the more active is the constitution in its attempts to resist the progress of such diseases, while at the same time a considerable portion of the general health and strength is consumed in the struggle.

Disease in the bony structure, and indeed of the teeth, without being influenced by any other causes than the local disorder itself, produce no greater constitutional effects than the local maladies; but with this difference, that their self-curative action is exerted in a proportion corresponding to the peculiar structure functions, and relation of those parts, and therefore comparatively much greater and longer continued than that produced by diseases of other parts or bones. In this they proceed very slowly, and their morbid effects can only be detected by the most minute attention.

---

